# Culture and Characterization of Microglia from the Adult Murine Retina

**DOI:** 10.1155/2014/894368

**Published:** 2014-05-29

**Authors:** Gayathri Devarajan, Mei Chen, Elizabeth Muckersie, Heping Xu

**Affiliations:** ^1^Infection and Immunity, School of Medicine, University of Aberdeen, Foresterhill AB25 2ZD, UK; ^2^Centre for Experimental Medicine, School of Medicine, Dentistry and Biomedical Sciences, Queen's University Belfast, Institute of Clinical Science-A, Grosvenor Road, Belfast BT12 6BA, UK

## Abstract

*Purpose*. To develop a protocol for isolating and culturing murine adult retinal microglia and to characterize the phenotype and function of the cultured cells. *Method*. Retinal single-cell suspensions were prepared from adult MF1 mice. Culture conditions including culture medium, growth factors, seeding cell density, and purification of microglia from the mixed cultures were optimised. Cultured retinal microglial cells were phenotyped using the surface markers CD45, CD11b, and F4/80. Their ability to secrete proinflammatory cytokines in response to lipopolysaccharide (LPS) stimulation was examined using cytometric bead array (CBA) assay. *Results*. Higher yield was obtained when retinal single-cell suspension was cultured at the density of 0.75 × 10^6^ cells per cm^2^ in Dulbecco's modified Eagle medium (DMEM)/F12 + Glutamax supplement with 20% fetal calf serum (FCS) and 20% L929 supernatant. We identified day 10 to be the optimum day of microglial isolation. Over 98% of the cells isolated were positive for CD45, CD11b, and F4/80. After stimulating with LPS they were able to secrete proinflammatory cytokines such as IL-6 and TNF-**α** and express CD86, CD40, and MHC-II. *Conclusion*. We have developed a simple method for isolating and culturing retinal microglia from adult mice.

## 1. Introduction

Microglia are specialised macrophages present in the retina and the central nervous system (CNS). They are derived from primitive progenitors in the yolk sac [1, 2]. Microglia constitute approximately 10% of the total glial cells in the CNS [3]. Microglia are vital for the healthy CNS and vision [4] and play an important role in retinal development, in injury, and during infection and repairing/regeneration [5]. Microglial activation is critically involved in several pathological conditions including age-related macular degeneration (AMD), Alzheimer's disease, and multiple sclerosis [6, 7]. Therefore, culturing microglia* in vitro* will be an important tool in understanding their pathophysiology and functions. Although the procedure for isolating and culturing brain-derived microglia is well established [8, 9], retinal microglia culture in particular from adult mice has proved to be more challenging.

Although the brain and retinal microglia have similar cell surface and enzymatic markers [9, 10], they live in different microenvironments and are associated with different pathophysiological conditions* in vivo* [6, 11]. Therefore, it is important to establish a reliable technique to culture retinal microglia for investigation of retinal diseases. The protocol for isolation and culture of retinal microglia from Royal College of Surgeons (RCS) rat has been established, and the cells were reported to be positive for CD11b, Griffonia simplicifolia isolectin B4, and vimentin [12]. However, the RCS rat has inherited retinal degeneration, and using the same protocol, the authors failed to produce retinal microglia with high yield from the normal rat retina [12]. There are a few recently published works on retinal microglia culture from the neonatal mice (postnatal days: 10–30) [13–15]. Weigelt and colleagues used retina from postnatal day 14 mice to isolate microglia [15]. The major issue in using microglia isolated from neonatal mice is that many of the cells are involved in the postdevelopment retinal remodelling process and have active phenotype (they rarely have a ramified morphology), whereas microglia in adult mice are well settled in the retina and have ramified morphology [16]. The phenotype and function of microglia from neonatal and adult mice differ significantly. For example, a study comparing brain microglia isolated from neonatal mice (postnatal day 8) and adult mice (6–8 weeks) revealed altered response to TLR-2, TLR-3, and TLR-4 agonists, lipopeptide PAM3CSK4, polyinosine-polycytidylic acid, and lipopolysaccharide, respectively (LPS) [16]. Microglia isolated from adult mice expressed high levels of cytokines such as TNF-*α*, IL-1*β*, and IL-6, whereas microglia from neonatal mice produced increased level of nitric oxide and expressed higher levels of inducible nitric oxide synthases (iNOS) after LPS and IFN-*γ* stimulation [16]. Most of the retinal diseases such as diabetic retinopathy and AMD occur in the adults/aged populations. Therefore, it is vital to use microglia from the adult mice to conduct pathophysiological investigations.

Using the protocol for culturing retinal microglia from neonatal mice, we were unable to yield sufficient number of cells from adult mice (8–12 weeks). Therefore, the aim of this study was to develop a microglia culture protocol for isolating high purity and a large number of microglia from adult mouse retina.

## 2. Methods

### 2.1. Isolation and Culture of Retinal Microglia

Male and female MF1 mice aged 2-3 months were purchased from Harlan Laboratories (Blackthorn, UK). The mice were housed at the University of Aberdeen animal facility under conditions of 12 h light and 12 h dark cycle, constant temperature, and free access to food and water. All the animal procedures were conducted under the regulation of the UK Home Office Animals (Scientific Procedures) Act 1986 and complied with the ARVO Statement for the Use of Animals in Ophthalmic and Vision Research.

The mice were sacrificed and eyes were removed and placed in ice-cold Dulbecco's modified Eagle medium (DMEM). The cornea and lens were removed and retinas were carefully dissected from the eye-cup under a microscope. Eight retinas were polled together and they were mechanically dissociated by harsh aspiration in culture medium. The tissues were then treated with 5 *μ*g/mL of collagenase type A (Sigma-Aldrich, Dorset, UK) in DMEM/F12 + Glutamax containing 10% fetal calf serum (FCS) and penicillin/streptomycin for 1 h at 37°C. The mixed retinal single-cell suspension was washed in DMEM/F12 + Glutamax and filtered through a 100 *μ*m cell strainer (BD Falcon, Biosciences, Oxford, UK). The retinal single-cell suspension was seeded in a 24-well plate (Cellstar Greiner Bio-one, Gloucestershire, UK) containing 20% FCS, 20% L929 conditioned medium, penicillin/streptomycin in DMEM, or RPMI (Roswell Park Memorial Institute)/F12 + Glutamax or DMEM/F12 + Glutamax. F12 + Glutamax was used to increase media stability, reduce ammonium, and provide more stable glutamine. After 10 days of culture, nonadherent cells were removed. The remaining adherent cells (i.e., microglial cells) were left to replicate further (2-3 weeks) in the 24-well plate (Nunc Thermo scientific, Leicestershire, UK).

Cell morphology was observed by phase-contrast microscopy throughout the experiment. Images were captured using a microscope (IMT-2; Olympus, Tokyo, Japan) fitted with a digital camera (Jenoptik Laser, Optik System GmbH, Jena, Germany). Images were processed using the ProgRes C14 Imaging software (Jenoptik Laser).

### 2.2. L929 Cell Culture

L929 is a mouse fibroblast cell line [17, 18]. The cells were cultured in DMEM supplemented with 10% FCS and penicillin/streptomycin. Supernatant from L929 is used in macrophage cultures as a source of macrophage-colony stimulating factor (M-CSF) [17, 18]. M-CSF promotes survival of macrophages and induces the differentiation of monocyte precursors. The L929 cells were grown in 75 cm^2^ cell culture flasks (Cellstar, Greiner Bio-one, Gloucestershire, UK) and were allowed to reach 90% confluency. The culture medium was then discarded and replaced with 20 mL of fresh medium and the conditioned medium was collected 72 h later. The supernatant was centrifuged, filtered, and stored at −80°C until use.

### 2.3. Immunofluorescent Staining

Retinal microglial cells were transferred into 16-well chamber slides (Labtek Nunc, Thermo scientific, Leicestershire, UK) and allowed to adhere for 24 h. Medium was discarded and cells were washed in phosphate buffer saline (PBS). Cells were blocked with 5% bovine serum albumin (Sigma-Aldrich, Dorset, UK) in PBS (Oxoid, Thermo scientific, Leicestershire, UK) for 30 min followed by streptavidin and biotin blocking step (Vector Laboratories Ltd., Peterborough, UK). Samples were incubated with primary antibody either purified or conjugated with biotin or fluorochrome for 1 h at room temperature. The antibodies used include purified rat anti-mouse CD45 (1 : 50, BD Biosciences, Oxford, UK), biotin conjugated anti-mouse CD11b (1 : 100, AbD Serotec, Kidlington, UK), biotin conjugated anti-mouse F4/80 (1 : 100, AbD Serotec), biotin conjugated anti-mouse CD40 (1 : 100, BD Biosciences), phycoerythrin (PE) conjugated anti-mouse CD86 (1 : 100, BD Biosciences), and fluorescein isothiocyanate (FITC) conjugated anti-mouse MHC-II (BD Biosciences). After the incubation, cells were washed in PBS, followed by incubation with one of below secondary antibodies: (1) biotin conjugated goat anti-rat IgG (1 : 200, BD Biosciences) for CD45; (2) streptavidin conjugated to Allophycocyanin (APC) (1 : 100, BD Biosciences) for biotinylated primary antibodies. Samples were incubated for 1 h at room temperature. Following the incubation with the secondary antibody conjugated to biotin for CD45, samples were incubated with Streptavidin APC as above. Samples were washed in PBS and mounted with the fluorescent mounting medium (Vectashield Vector Laboratories, Peterborough, UK) and slides were examined by confocal microscopy (LSM5110 META; Carl Zeiss, Jena, Germany).

### 2.4. Isolation and Labelling of Photoreceptor Outer Segment (POS)

*Isolation*. POS were isolated from bovine eyes according to the method of Molday and Molday (1987) [19]. Isolated POS were resuspended in storage buffer (10 mM sodium phosphate, pH 7.2, 0.1 M NaCl, and 2.5% sucrose) at the density of 1 × 10^8^/mL and stored at −80°C until use. 

*Labelling*. POS were washed in PBS and incubated with FITC (Invitrogen, UK) dissolved in DMSO with a final concentration of 2 mg/mL. POS were incubated with 100 *μ*L of FITC in 400 *μ*L of sterile sodium bicarbonate buffer (0.1 M sodium bicarbonate pH 9) for 1 h in the dark at room temperature. After the incubation, POS were washed in PBS 4 times.

### 2.5. Phagocytosis of POS-FITC by Microglia

Retinal microglial cells were transferred into 16-well chamber slides and cultured for 24 h. These cells were incubated with POS-FITC at the ratio of cells to POS-FITC 1 : 5 for different times (30 min, 1 h, 2 h, 4 h, 6 h, 12 h, and 18 h). The cells were fixed and stained for CD11b (BD Biosciences) as mentioned above.

### 2.6. Cytokine and Chemokine Measurement

Microglial cells were transferred into 16-well chamber slides and cultured for 24 h. Medium was discarded and the cells were washed in PBS and incubated with 1 *μ*g/mL of LPS (Sigma-Aldrich, Dorset, UK) in the culture medium for 24 h. The supernatants were collected and measured for the presence of cytokines and chemokines including TNF-*α*, IL-1, IL-12, IL-6, CCL-5, CCL-2, GM-CSF, and IL-10 using the cytometric bead array (CBA) kit. CBA was performed according to the manufacturer's instructions. Flat bottom 96-well plate (Nunc, Thermo scientific, Leicestershire, UK) was coated with capture beads and the standards and samples were added to the appropriate wells and incubated for 1 h at room temperature in the dark. This was followed by the incubation with PE detecting reagent for 1 h at room temperature in the dark. The plate was aspirated using vacuum manifold and the beads were washed with the washing buffer and samples were analysed using FACS Array (BD Biosciences).

### 2.7. Statistical Analysis

Statistical analyses were performed using the Graphpad software (Graphpad, San Diego, CA, USA). Comparisons between 3 or more data groups were performed using one-way analyses of variance (ANOVA), and comparisons between 2 groups were performed using two-tailed Student's *t*-test. A *P* value <0.05 was set as the basis for rejecting the null hypothesis (i.e., the group means being compared do not differ significantly from each other). In all graphical representations the error bars indicate standard error of the mean (SEM).

## 3. Results

### 3.1. Optimising Microglia Culture Conditions

**Figure 1:**
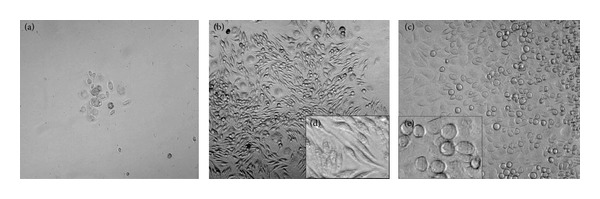
Effects of different culture media on the yield and the morphology of the cultured microglial cells. (a) Mixed retinal culture grown in DMEM showing low cell numbers. ((b) and (d)) Cells grown in DMEM/F12 + Glutamax showing a good yield and no change in the morphology was observed. ((c) and (e)) Some of the cells grown in RPMI/F12 + Glutamax started to change into more round-shaped cells. Microglial cells were observed after 15 days in culture using a phase-contrast Olympus microscope and Prog Res C14 imaging software. Magnification ×20; (d) and (e) are enlarged. Data are representative of 3 experimental repeats.

*Culture Medium*. To identify the media that are best suited for microglial cells, we tested DMEM, RPMI/F12 + Glutamax or DMEM/F12 + Glutamax. The mixed retinal cells were seeded in a 24-well plate at the density of 0.75 million cells per cm^2^ and supplemented with FCS and L929 supernatant. Cells cultured in DMEM failed to proliferate (Figure 1(a)), whereas cells grown in DMEM/F12 + Glutamax and RPMI/F12 + Glutamax proliferated (Figures 1(b) and 1(c), resp.). Microglial cells cultured in DMEM/F12 + Glutamax resembled a more ramified shape, whereas cells grown in RPMI/F12 + Glutamax have macrophage-like amoeboid morphology (Figures 1(d) and 1(e), resp.). Therefore, DMEM/F12 + Glutamax provides an optimum condition for the growth and maintenance of retinal microglia.

**Figure 2:**
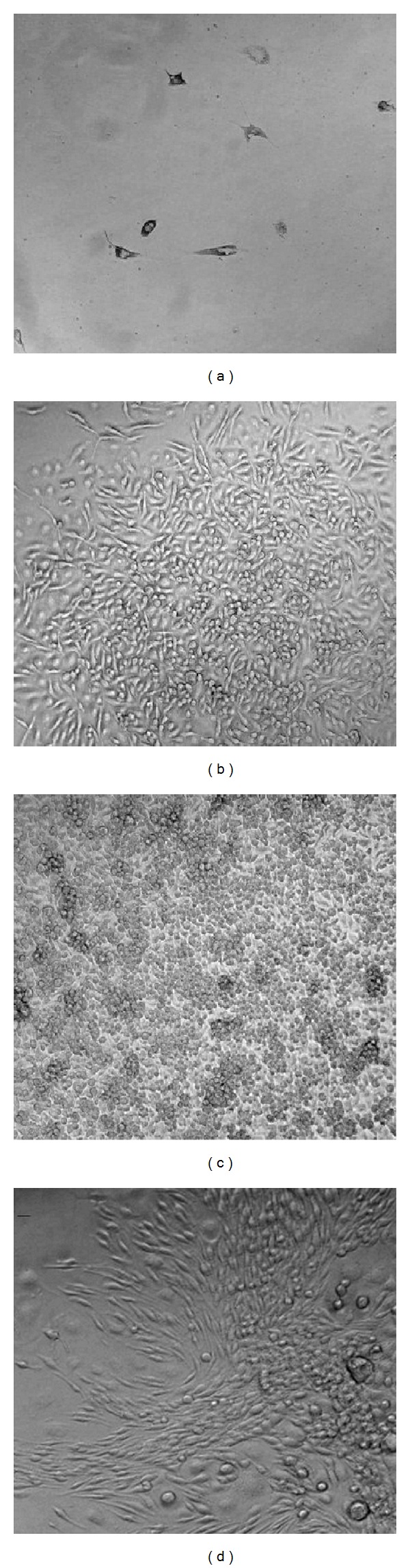
Morphology and proliferation of the cultured microglial cells in different percentages of the L929 conditioned supernatant. (a) Mixed retinal culture grown in 0% L929 supernatant yielded few cells. (b) 10% and (c) 20% of L929 conditioned supernatant produced the highest yield. (d) 30% L929 conditioned supernatant produced less yield compared to 20%. Microglial cells were observed using a phase-contrast Olympus microscope and Prog Res C14 imaging software. Magnification ×20. Data are representative of 3 experimental repeats.

The major problems facing retinal microglia culture are limited cell numbers and low proliferation rate. To induce proliferation of the isolated microglia we used L929 supernatant. L929 fibroblast cells produce various growth factors including M-CSF [18]. To optimise cell proliferation, the culture medium was supplemented with varying percentages of L929 supernatant. Microglial cells cultured without L929 supernatant failed to proliferate (Figure 2(a)). Microglial cells proliferated in response to L929 supernatant and we observed cells supplemented with 20% L929 supernatant in DMEM/F12 + Glutamax to be the optimal condition (Figure 2(c)). 

**Figure 3:**
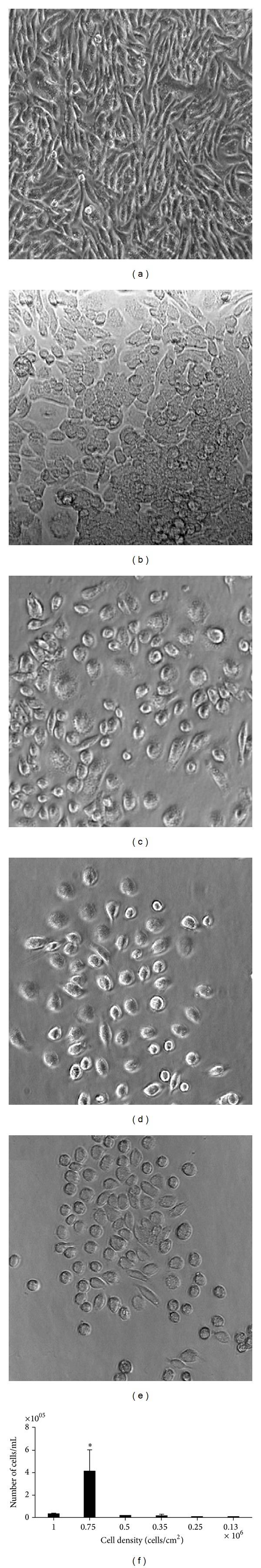
Influence of initial seeding cell densities on the microglia culture. Mixed retinal cells seeded at 0.75–1 × 10^6^ cells/cm^2^ produced the highest cell numbers. (a) 1 × 10^6^ cells/mL. (b) 0.75 × 10^6^ cells/mL. (c) 0.5 × 10^6^ cells/mL. (d) 0.25 × 10^6^ cells/mL. (e) 0.13 × 10^6^ cells/mL. After 15–20 days of culture, retinal microglial cells were observed using a phase-contrast Olympus microscope and Prog Res C14 imaging software. Magnification ×20. Cells were counted using haemocytometer and trypan blue was used to distinguish viable and dead cells. Error bars are SEM. *P < 0.05, compared to others groups.

*Cell Density*. Microglial cells constitute a minor percentage of the total cells in retinal single-cell suspension. This resulted in the requirement for the optimal initial seeding density. The cell densities utilised in this study ranged from 0.13 × 10^6^ to 1 × 10^6^ cells per cm^2^ (Figure 3). The results showed that 0.75 × 10^6^ cells per cm^2^ gave higher yield consistently compared to other densities tested (Figure 3(f)) and, therefore, were selected for further cultures of the study.

### 3.2. Timing of Removing Nonmicroglial Cells

**Figure 4:**
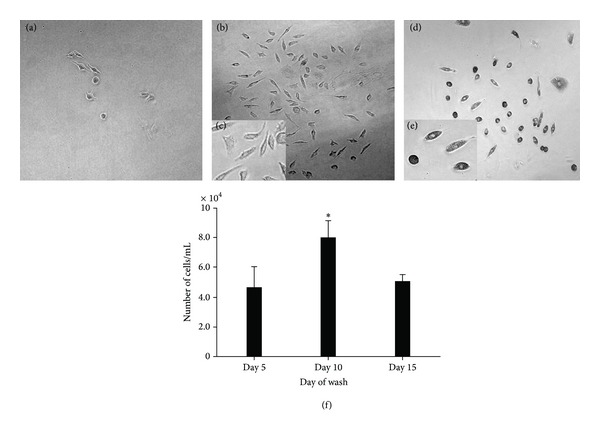
Microglial cells isolated from the mixed culture on different days influence the microglial cell numbers and morphology. (a) Removing nonadherent cells on day 5 yielded the least number of cells. ((b) and (c)) Microglial cultures on day 10. ((d) and (e)) Microglial cultures on day 15. Day 10 seems to be ideal time to wash away loosely adherent cells and dead cells. Day 15 seems to affect the morphology of the cell. Microglial cells were observed using a phase-contrast Olympus microscope and Prog Res C14 imaging software. Magnification ×20. (f) Microglial cells were counted using haemocytometer after different days of the initial seeding. Error bars are SEM for 2 replicate cultures. *P < 0.05 compared to day 5 and day 15.

In the presence of L929 supernatant, microglial cells were able to survive and attach to culture plates, whereas other cells such as photoreceptors, neurons, and other glial cells were unable to survive due to the lack of proper growth factors. The dead cells need to be removed from the culture promptly, as they may be toxic to other live cells. To identify the optimum time scale for the microglial cells to attach and settle in their new environment, nonadherent cells and dead cells were removed from the culture on day 5, day 10, or day 15 after seeding. At day 5 (Figure 4(a)), very few microglial cells were yielded compared to days 10 and 15 (Figures 4(b) and 4(d), resp.). However, on day 15 the morphology of the cells appeared to be rounded, with increased granules present inside the cells (Figure 4(e)). This may indicate that microglia were phagocytosing cell debris. Therefore, day 10 appeared to be an appropriate point to remove loosely adherent and dead cells from the culture and isolate the microglia for the continuation of the culture. The morphology of the cells was consistent with those previously observed. The number of cells yielded at day 10 was higher compared to other time points (Figure 4(f)). Nonadherent and loosely adherent cells were removed by gentle aspiration without the need of trypsinization [20]. Presence of trypsin can affect the viability of the cells; therefore, microglia can be isolated without harming the cells.

The data presented here are representative of the optimised culture conditions for murine adult retinal microglia. We have demonstrated that the proliferation of morphologically defined microglia is optimal at the initial seeding density of 0.75 × 10^6^ cells per cm^2^ in DMEM/F12 + Glutamax supplemented with 20% of L929 conditioned supernatant and cellular isolation on day 10.

### 3.3. Phenotype of Cultured Retinal Microglial Cells

**Figure 5:**
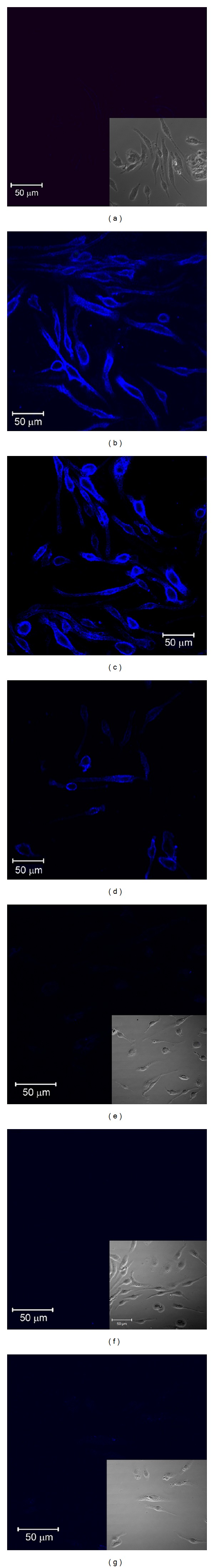
Immunofluorescent staining of cultured retinal microglial cells. Microglial cells were transferred into a 16-well chamber slide and stained for (a) isotype control (insert, phase contrast image to show the cells), (b) F4/80, (c) CD11b, (d) CD45, (e) MHC-II (insert, phase contrast), (f) CD86 (insert, phase contrast), and (g) CD40 (insert, phase contrast). The samples were analysed by confocal microscopy. In all images blue indicates allophycocyanin (APC). Over 98% of the cells were positive for CD11b, CD45, and F4/80 surface markers but the staining of CD45 was much weaker compared to CD11b and F4/80. Cells were negative for MHC-II, CD86, and CD40. Data were representative of 2 experiments.

**Figure 6:**
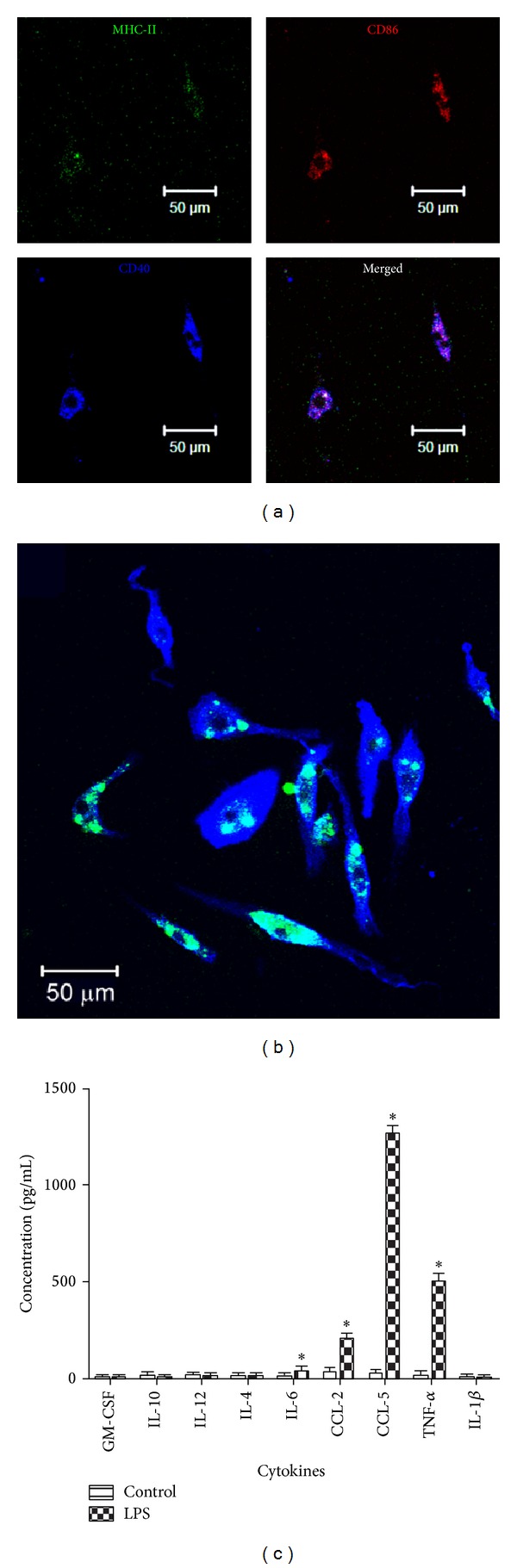
Immunofluorescent staining of retinal microglial cells. (a) Microglial cells were transferred into a 16-well chamber slide and stimulated with LPS in the absence of L929 supernatant for 24 h. After stimulation, cells were stained for activation markers, including MHC-II (FITC), CD86 (PE), and CD40 (APC). The slides were observed by confocal microscopy. (b) Microglial cells were transferred into a 16-well chamber slide, incubated with POS-FITC at the ratio of cells to POS-FITC 1 : 5 for 18 h, and stained for CD11b (APC). (c) Microglial cells (1 × 10^4^/well) were transferred into a 96-well plate and stimulated with 1 *μ*g/mL of LPS for 24 h. The supernatant was collected and measured for the presence of IL-1*β*, IL-12, IL-10, CCL2, GM-CSF, IL-6, TNF-*α*, and CCL5 using CBA. *P ≤ 0.05 compared with control nonstimulated cell supernatant. Mean ± SEM, *n* = 3.

To characterise the cells, the retinal cultures were transferred into a chamber slide and stained for the surface markers including CD11b, F4/80, and CD45. None of the above markers are specific to the retinal microglial cells. However, microglia are generally characterised as CD11b^high^ and CD45^low^. The majority (>98%) of the cultured cells were positive for CD11b indicating that the cells belong to the mononuclear phagocytic system. The cells expressed high levels of F4/80 (Figure 5(b)) and CD11b (Figure 5(c)) and low levels of CD45 (Figure 5(d)). The cultured retinal microglial cells were negative for MHC-II, CD86, and CD40 (Figures 5(e), 5(f), and 5(g), resp.) indicating that they were not activated. However, microglial cells were positive for MHC-II, CD86, and CD40 in response to LPS stimulation (Figure 6(a)).

### 3.4. Functions of the Microglial Cells

*Phagocytosis.* One of the main functions of retinal microglia is to phagocytise cell debris. It is important to identify if* in vitro* cultured microglial cells have the ability to maintain this function. Macrophages are known to phagocytise POS through *α*v*β*3 integrin [21]. FITC-conjugated POS were incubated with the cultured microglial cells. After 8 h incubation, 75% of CD11b^+^ microglial cells were positive for POS-FITC (Figure 6(b)). 

*Cytokine and Chemokine Production.* To ensure that the cultured cells can respond to stimulation as would be expected* in vivo*, we investigated the cytokine production in response to LPS. Microglial cells were transferred into a 96-well plate and stimulated with 1 *μ*g/mL of LPS for 24 h. The supernatant was analysed for the presence of cytokines and chemokines. The cells produced significant amount of IL-6, CCL2, TNF-*α*, and CCL5 upon LPS stimulation (Figure 6(c)). The production of IL-4, IL-10, IL-12, and GM-CSF was low in both resting and LPS-stimulated cells (Figure 6(c)).

## 4. Discussion

The problems facing murine retinal microglia culture are limited cell numbers, low proliferation rate, and isolation of microglial cells from the mixed glial population. A recent study by Weigelt and colleagues (2007) [15] has proposed a method for the isolation and culture of retinal microglia from 14-day postnatal C57BL/6 mice, using density gradient centrifugation followed by culture in DMEM with 10% FCS and 50 ng/mL of recombinant M-CSF [13, 15]. However, this protocol was not suitable for adult mouse microglial culture.

We found that cells cultured in DMEM/F12 + Glutamax had better morphology and proliferate better compared to those cultured in DMEM; this was surprising because brain microglia and neonatal retinal microglia were able to grow in DMEM [9, 15]. This is probably related to the higher number of microglia present in the brain tissue compared to that of a retinal tissue, and neonatal retinal microglia have higher proliferation rate compared to adult microglia. DMEM/F12 + Glutamax media contain stable glutamine and provide a more steady and ideal growth conditions to low number of retinal microglial cells in the mixed culture.

Morphologically these retinal microglia appear similar to the brain microglia [9, 15]. The majority of retinal microglial cells cultured in DMEM/F12 + Glutamax had long and slender cell body, and a very small population of cells were round-shaped, whereas the majority of the microglial cells grown in RPMI/F12 + Glutamax were round-shaped similar to macrophages. This suggests that these isolated microglial cells are versatile and have the ability to change in response to their environment.

We utilised L929 supernatant, which has been demonstrated to be a potent source of M-CSF and has previously been shown to increase the proliferation and survival of microglia [22]. The conditioned supernatant has also been used to grow brain microglia and the proliferation of the microglia was dependent on the percentage of the crude M-CSF present in the culture medium. However, higher concentration such as 30% or above had a negative impact on the proliferation acting to prevent excessive cell growth. In addition to the excess growth factors, the increase percentage of L929 supernatant also diminished the percentage of the complete medium, which contained supplemented growth nutrients. The retinal cells required a higher initial seeding density to obtain a substantial yield. Microglial cells proliferated better when they were in contact with each other. This protocol requires minimum of four retinas to obtain a good yield.

Retinal microglial cells were purified from the mixed culture by removing loosely adherent and dead cells. The time to remove nonadherent, loosely adherent, or dead cells is crucial. We observed higher number of microglial cells present on days 10 and 15. However, on day 15 microglial cells appeared morphologically active due to the presence of prolonged dead cells in the culture media and this resulted in increased phagocytic activity in the microglial cells to clear dead cells from the surrounding microenvironment. We observed that this reduces their ability to proliferate further in the culture.

The cultured retinal microglial cells appear to maintain their functions* in vitro*. They can phagocytise POS with large numbers. They are also able to produce inflammatory cytokines and chemokines such as IL-6 and TNF-*α* and express various activation markers, CD40, CD86, and MHC-II upon LPS stimulation.

In summary, in this study we developed a simple and reliable method to isolate and culture microglial cells from the adult murine retina. The protocol allows growing sufficient number of cells from as few as 8 retinas for various* in vitro* studies. More importantly, cells cultured using this protocol maintained the key phenotype and function of retinal microglial cells.
